# IL-10 mediated by herpes simplex virus vector reduces neuropathic pain induced by HIV gp120 combined with ddC in rats

**DOI:** 10.1186/1744-8069-10-49

**Published:** 2014-07-30

**Authors:** Wenwen Zheng, Wan Huang, Shue Liu, Roy C Levitt, Keith A Candiotti, David A Lubarsky, Shuanglin Hao

**Affiliations:** 1 Department of Anesthesiology, University of Miami Miller School of Medicine, 1550 NW 10th Ave, Fox BLDG, Rm 304C, Miami, FL 33136, USA; 2 Hussman Institute of Human Genomics, University of Miami Miller School of Medicine, Miami, FL 33136, USA; 3 Veterans Affairs Medical Center, Miami, FL 33136, USA

**Keywords:** HIV, Neuropathic pain, gp120, ddC, IL-10, and Gene therapy

## Abstract

**Background:**

HIV-associated sensory neuropathy affects over 50% of HIV patients and is a common peripheral nerve complication of HIV infection and highly active antiretroviral therapy (HAART). Evidence shows that painful HIV sensory neuropathy is influenced by neuroinflammatory events that include the proinflammatory molecules, MAP Kinase, tumor necrosis factor-α (TNFα), stromal cell-derived factor 1-α (SDF1α), and C-X-C chemokine receptor type 4 (CXCR4). However, the exact mechanisms of painful HIV sensory neuropathy are not known, which hinders our ability to develop effective treatments. In this study, we investigated whether inhibition of proinflammatory factors reduces the HIV-associated neuropathic pain state.

**Results:**

Neuropathic pain was induced by peripheral HIV coat protein gp120 combined with 2′,3′-dideoxycytidine (ddC, one of the nucleoside reverse transcriptase inhibitors (NRTIs)). Mechanical threshold was tested using von Frey filament fibers. Non-replicating herpes simplex virus (HSV) vectors expressing interleukin 10 (IL10) were inoculated into the hindpaws of rats. The expression of TNFα, SDF1α, and CXCR4 in the lumbar spinal cord and L4/5 dorsal root ganglia (DRG) was examined using western blots. IL-10 expression mediated by the HSV vectors resulted in a significant elevation of mechanical threshold. The anti-allodynic effect of IL-10 expression mediated by the HSV vectors lasted more than 3 weeks. The area under the effect-time curves (AUC) in mechanical threshold in rats inoculated with the HSV vectors expressing IL-10, was increased compared with the control vectors, indicating antinociceptive effect of the IL-10 vectors. The HSV vectors expressing IL-10 also concomitantly reversed the upregulation of p-p38, TNFα, SDF1α, and CXCR4 induced by gp120 in the lumbar spinal dorsal horn and/or the DRG at 2 and/or 4 weeks.

**Conclusion:**

The blocking of the signaling of these proinflammatory molecules is able to reduce HIV-related neuropathic pain, which provide a novel mechanism-based approach to treating HIV-associated neuropathic pain using gene therapy.

## Background

In Europe and the U.S., approximately 2-7% of the population suffers from neuropathic pain, a condition caused by damage and/or inflammation to nerves following traumatic injury, viral infection or chemotherapy [1]. Neuropathic pain (NP) related to human immunodeficiency virus-1 (HIV) infection is severe and unrelenting and represents an important unmet need in medicine [2]. This HIV neuropathy can be associated with viral infection alone, probably involving a role for the envelope glycoprotein gp120 and/or antiretroviral drug-induced toxic neuropathy associated with the use of nucleoside analogue reverse transcriptase inhibitors as a component of highly active anti-retroviral therapy (HAART). Moreover, a growing body of evidence demonstrates that proinflammatory mediators including tumor necrosis factor alpha (TNFα) released by the activated spinal glial cells and in the dorsal root ganglia (DRG), are critical to enhancing pain [3-8]. Activation of p38 mitogen-activated protein kinase (MAPK) is known to be important in cytokine regulation. The expression of phospho-p38 (p-p38) in the DRG is well- characterized following peripheral nerve injury associated with pathological pain [9,10]. The C-X-C chemokine receptor type 4 (CXCR4) acts as an important pro-inflammatory factor in the neuropathogenesis of HIV/AIDS [11,12]. Furthermore, CXCR4 is required for gp120-induced cell death [13]. Importantly, our studies and others show that stromal cell-derived factor-1α (SDF1α) and its receptor CXCR4 are involved in the NP induced by gp120 [14] or ddC [15-17].

Interleukin 10 (IL-10) is an anti-inflammatory molecule that has achieved interest as a therapeutic for neuropathic pain. IL-10 blocks phosphorylation of MAPK pathways [18], and suppresses the production and function of many proinflammatory cytokines released by activated glia [19,20]. IL-10 diminishes the levels of TNF mRNAs after the onset of stimulation of polymorphonuclear leukocytes (PMN) with lipopolysaccharide (LPS), a very common proinflammatory inducer [21,22]. Lumbosacral intrathecal administration of the IL-10 transgene or protein leads to robust suppression of tactile allodynia induced by sciatic nerve injury, as well as spinal inflammation following a single intrathecal injection of gp120 protein [23-25]. Additionally, hyperalgesic responses to TNFα or carrageenan are inhibited by intraplantar administration of IL-10 (12).

While intrathecal IL-10 protein has been shown to provide relief from pain in animal models, these effects are short and closely parallel the half-life of IL-10 in the cerebrospinal fluid (CSF) [25]. To provide prolonged pain relief, gene therapy vectors encoding for the production of IL-10 have been utilized [8,23,25]. The highly replication-defective herpes simplex virus (HSV) genomic vectors can establish a persistent state that is used to deliver and express transgenes in DRG neurons. DRG neurons transduced with the HSV vector transport transgene-coded enkephalin centrally in the bipolar axon of the primary sensory afferents to the spinal dorsal horn (SDH) [26]. Inoculation of the HSV vectors expressing the IL-10 gene significantly reduces mechanical allodynia below the level of injury after blunt trauma to the spinal cord [27]. HIV neuropathic pain with a sizeable morbidity is difficult to be treated in clinic [2]. In this study, we tested the hypothesis that HSV vector-mediated IL-10 expression could treat NP induced by HIV gp120 with antiretroviral drug. We found that this IL-10 gene therapy produced the antinociceptive effects on NP induced by the gp120 application with antiretroviral drug, and reduced proinflammatory molecules p-p38, TNFα, and CXCR4/SDF1α in the DRG and/or the SDH.

## Results

### The anti-allodynic effect of IL10 mediated by HSV vectors in the gp120 +ddC model

**Figure 1:**
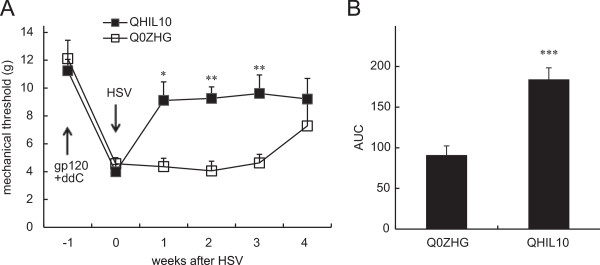
**The anti-allodynic effect of IL-10 mediated by the HSV vectors on neuropathic pain induced by HIV gp120 combined with ddC. (A)** Mechanical allodynia in rats was shown 1 week post the gp120 application with ddC. The times of gp120 + ddC and HSV vector inoculation were indicated by arrows. QHIL10 resulted in a statistically significant elevation of the mechanical threshold (g) compared with the control vectors (*F*_(1,12)_ = 11.996, *P* < 0.01, repeated measures ANOVA, n = 7). The comparison of differences at individual time points between two groups was shown, *P < 0.05, **P < 0.01 *vs.* Q0ZHG, two-tailed *t* test. **(B)** The AUC in QHIL10 group was significantly higher than that in Q0ZHG, ***P < 0.001, *t* test, n = 7 rats).

Previous studies have demonstrated that the peripheral gp120 application into the sciatic nerve, systemic ddC, or combination of gp120 and ddC (gp120+ddC), results in neuropathic pain characterized by mechanical allodynia and upregulates TNFα [4,28-31]. The principal anti-inflammatory activity of IL-10 is to inhibit the production of proinflammatory cytokines [21]. We have demonstrated that IL-10 mediated by the HSV vectors reversed formalin-induced inflammatory pain [8]. Recent studies show that animals inoculated with the HSV vectors expressing IL10 reduces mechanical allodynia induced by the spinal cord injury [27]. In this study, we further examined whether overexpression of IL-10 mediated by the HSV vectors, reduced neuropathic pain induced by HIV gp120 + ddC. HIV gp120 combined with ddC induced a rapid decrease in mechanical threshold at 1 week. Subcutaneous inoculation with QHIL10 (30 μl containing 1 × 10^9^ plaque-forming units/ml) or control vector Q0ZHG was carried out in the plantar surface of the hind foot of rats with neuropathic pain 1 week post application of gp120 + ddC. QHIL10 resulted in a statistically significant elevation of mechanical threshold that was apparent on day 7 post vector inoculation compared with the control vectors (*F*_(1,12)_ = 11.996, *P* < 0.01, repeated measures ANOVA, n = 7) (Figure 1A). The anti-allodynic effect of the HSV vectors lasted for more than 3 weeks. For the comparison of the differences at individual time points between two groups, we used a two-tailed *t* test; there was a significant difference at week 1, 2, and 3 between the 2 groups. The area under the effect-time curves (AUC) after HSV in the QHIL10 group was significantly higher than that in the Q0ZHG group (*P* < 0.001, *t* test, n = 7, Figure 1B).

### The effect of HSV vectors over-expressing IL-10 on p-p38 in the DRG and the SDH in the gp120 + ddC model

**Figure 2:**
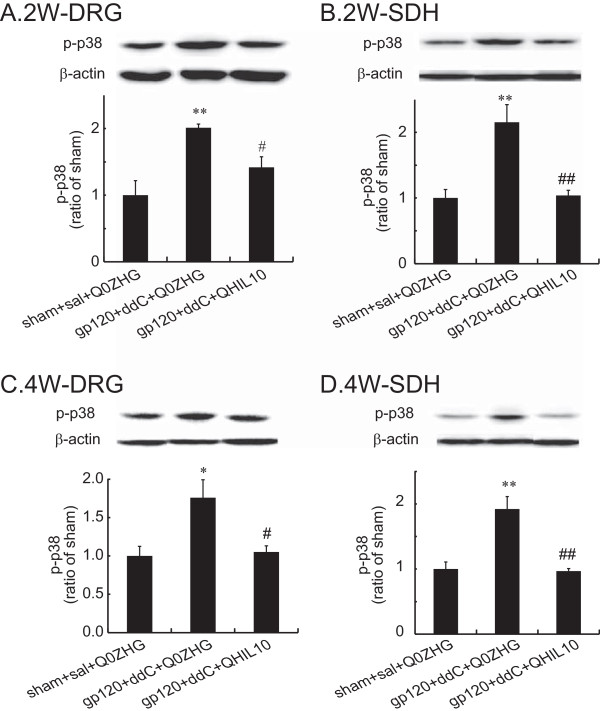
**The effect of IL-10 mediated by the HSV vectors on the expression of p-p38 in the DRG and the SDH at 2 or 4 weeks.** Rats with neuropathic pain were inoculated with QHIL10 or Q0ZHG 1 week post gp120 application with ddC. In the control group, rats received the sham surgery with saline IP injection and Q0ZHG (sham + sal + Q0ZHG). **(A and B)** Two weeks post vector injection, the L4/5 DRG **(A)** and the SDH **(B)** were harvested, and the expression of p-p38 was tested using western blots. The data were analyzed using one way ANOVA with *post hoc* PLSD test, mean ± SEM, **P < 0.01 *vs.* control group, # P < 0.05, ## *P* < 0.01 *vs* gp120 + ddC + Q0ZHG, n = 4 rats. **(C and D)** Four weeks post vector injection, the L4/5 DRG **(C)** and the SDH **(D)** were harvested, and the expression of p-p38 was tested using western blots. The data were analyzed using one way ANOVA with *post hoc* PLSD test, mean ± SEM, *P < 0.05, **P < 0.01 *vs.* control, # *P* < 0.05, ## *P* < 0.01 *vs* gp120 + ddC + Q0ZHG, n = 4 rats.

Activated MAP kinase p-p38 plays important role in the maintenance of inflammatory/neuropathic pain [3,8,32]. In this study, we investigated whether the over-expression of IL10 mediated by the HSV vectors reduced p-p38 in the gp120 + ddC model. The L4/5 DRG and the SDH were harvested on 2 weeks post vector injection. The pooled L4/5 DRG and the SDH were used for western blots. The data were presented as mean ± SEM and were compared using one way ANOVA with a *post hoc* PLSD test (StatView), n = 4 rats. In the DRG samples 2 weeks post vector injection, neuropathic rats inoculated with Q0ZHG showed a statistically significant increase in the expression of p-p38 compared with that in the sham with Q0ZHG (*P <* 0.01 *vs* sham + sal + Q0ZHG, Figure 2A); the expression of p-p38 in neuropathic rats with QHIL10 was markedly lower than that with Q0ZHG (*P <* 0.05 *vs* gp120 + ddC + QOZHG, Figure 2A). In the SDH samples 2 weeks post vector injection, the expression of p-p38 in neuropathic rats with Q0ZHG was markedly increased compared with that in sham rats (*P <*0.01 *vs* sham + sal + Q0ZHG, Figure 2B); p-p38 in neuropathic rats with QHIL10 was lower than that with Q0ZHG (*P <*0.01 *vs* gp120 + ddC + QOZHG, Figure 2B).

Similarly, in a separate set of experiments, at 4 weeks post vector injection, the L4/5 DRG and the SDH were harvested for western blots. In the DRG, there was a significant increase in p-p38 in neuropathic rats with Q0ZHG compared with that in sham rats with Q0ZHG *(P* <0.05 *vs* sham + sal + Q0ZHG, Figure 2C); the expression of p-p38 in neuropathic rats with QHIL10 was significant lower than that in neuropathic rats with Q0ZHG (*P* <0.05 *vs* gp120 + ddC + QOZHG, Figure 2C). In the SDH samples 4 weeks post vector injection, p-p38 in neuropathic rats with Q0ZHG was markedly increased compared with that in the sham group (*P* <0.01 *vs* sham + sal + Q0ZHG, Figure 2D); in neuropathic rats treated with QHIL10, p-p38 was lower than that with Q0ZHG (*P* < 0.01 *vs* gp120 + ddC + Q0ZHG, Figure 2D).

### The effect of HSV vectors over-expressing IL-10 on TNFα in the DRG and the SDH in the gp120 + ddC model

**Figure 3:**
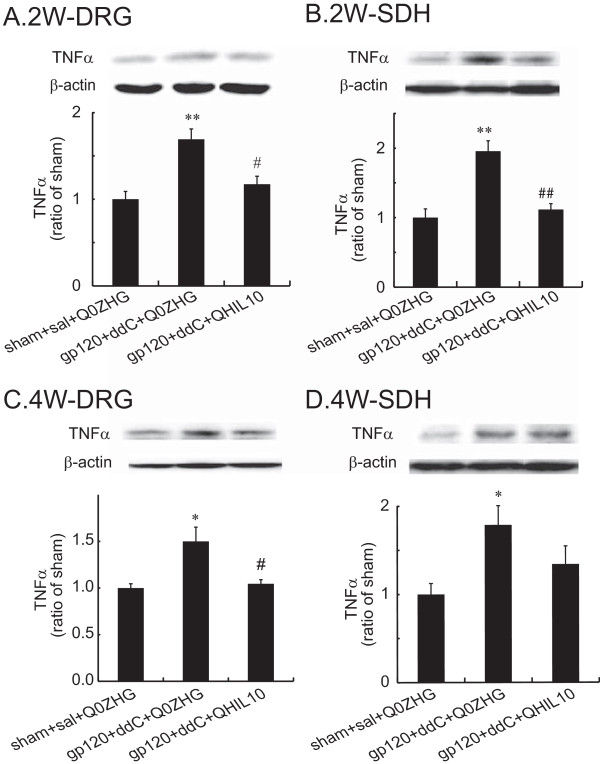
**The effect of IL-10 mediated by the HSV vectors on the expression of TNFα in the DRG and the SDH at 2 or 4 weeks.** Rats with neuropathic pain were inoculated with QHIL10 or Q0ZHG 1 week post gp120 with ddC. In the control group, rats received the sham surgery with saline IP injection and Q0ZHG (sham + sal + Q0ZHG). The data were analyzed using one way ANOVA with *post hoc* PLSD test, mean ± SEM. **(A and B)** Two weeks post vector injection, the L4/5 DRG **(A)** and the SDH **(B)** were harvested, and the expression of TNFα was tested using western blots. ** *P <* 0.01 *vs.* control, # *P <* 0.05, ## *P* < 0.01 *vs* gp120 + ddC + Q0ZHG, n = 4 rats. **(C and D)** Four weeks post vector injection, the L4/5 DRG **(C)** and the SDH **(D)** were harvested, and the expression of TNFα was tested using western blots. *P < 0.05 *vs.* control, # *P* < 0.05 *vs* gp120 + ddC + Q0ZHG, n = 4 rats.

Evidence shows that HIV gp120 or ddC mediated-neuropathic pain increases TNFα in the spinal cord and the DRG [4,14,30]. In the current study, we examined whether overexpression of IL10 mediated by the HSV vectors reduced TNFα in neuropathic pain induced by gp120 + ddC. The L4/5 DRG and the SDH were harvested for western blots for full-length membrane TNFα on 2 weeks post vector injection. The data were presented as mean ± SEM, and were compared using one way ANOVA with a *post hoc* PLSD test (StatView), n = 4 rats. In the DRG, there was a marked increase in TNFα in the gp120 + ddC + Q0ZHG group compared with that in the sham group (*P* < 0.01 *vs* sham + sal + Q0ZHG, Figure 3A); TNFα in the gp120 + ddC + QHIL10 group, was significantly lower than that in the gp120 + ddC + Q0ZHG group (*P* < 0.05, Figure 3A). In the SDH, there was a marked increase in TNFα in the gp120 + ddC + Q0ZHG group compared with that in the sham group (*P* < 0.01, Figure 3B); TNFα expression in the gp120 + ddC + QHIL10 group was significantly lower than that in the gp120 + ddC + Q0ZHG (*P* < 0.01, Figure 3B).

In similar studies, at 4 weeks post vector injection, the L4/5 DRG and the SDH were harvested for western blots. In the DRG, there was a significant increase in TNFα in the group of gp120 + ddC + Q0ZHG compared with that in the sham group (*P* <0.05, Figure 3C); the increased TNFα was reversed by treatment with QHIL10 in the DRG (*P* <0.05, Figure 3C). In the SDH samples 4 weeks post vector injection, there was a marked increase in TNFα in gp120 + ddC + Q0ZHG group compared with that in sham group, however, there was no significant difference between gp120 + ddC + Q0ZHG group and gp120 + ddC + QHIL10 group (Figure 3D).

### The effect of HSV vectors over-expressing IL-10 on SDF1α in the DRG and the SDH in the gp120 + ddC model

**Figure 4:**
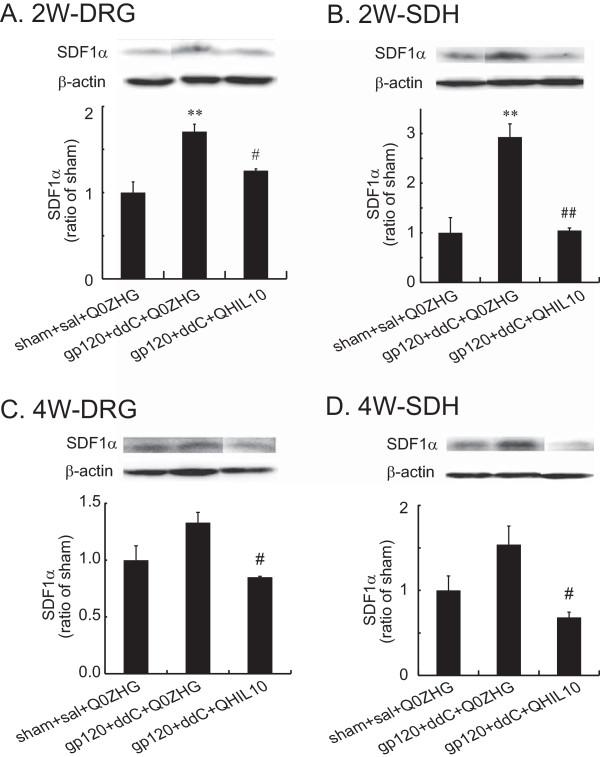
**The effect of IL-10 mediated by HSV vectors on the expression of SDF1α in the DRG and the spinal cord at 2 or 4 weeks.** Rats with neuropathic pain were inoculated with QHIL10 or Q0ZHG 1 week post gp120 with ddC. In the control group, rats received the sham surgery with saline IP injection and Q0ZHG (sham + sal + Q0ZHG). The data were analyzed using one way ANOVA with *post hoc* PLSD test, mean ± SEM. **(A and B)** Two weeks post vector injection, the L4/5 DRG **(A)** and the SDH **(B)** were harvested, and the expression of SDF1α was tested using western blots. ** *P* < 0.01 *vs*. control, # *P* < 0.05, ## P < 0.01 vs gp120 + ddC + Q0ZHG, n = 4 rats. **(C and D)** Four weeks post vector injection, the L4/5 DRG **(C)** and the SDH **(D)** were harvested, and the expression of SDF1α was tested using western blots. # *P* < 0.05 *vs.* gp120 + ddC + Q0ZHG, n = 4 rats.

TNFα enhances the expression of CXCR4, which facilitates the chemotactic invasiveness of the cultured human mesenchymal stem cells toward SDF1α [33]. We have reported that IL-10 is able to suppress overexpression of mRNA and protein of TNFα induced by formalin into the hindpaws [8]. However, it is not known if IL10 reduced production of SDF1α *in vivo* in the gp120 + ddC-induced neuropathic pain state. In this study, we investigated whether the overexpression of IL10 mediated by the HSV vectors reduced SDF1α in the neuropathic pain state. In the DRG samples 2 weeks post vector injection, there was a significant increase in SDF1α in neuropathic rats with Q0ZHG compared with that in sham rats (*P* < 0.01, Figure 4A); the expression of SDF1α in the DRG in neuropathic rats with QHIL10, was markedly lower than that in neuropathic rats with Q0ZHG (*P* < 0.05, Figure 4A). In the SDH samples 2 weeks post vector injection, there was a significant increase in SDF1α in neuropathic rats with Q0ZHG compared with that in the sham rats (*P* < 0.01, Figure 4B); expression of SDF1α in neuropathic rats treated with QHIL10 was markedly lower than that in neuropathic rats with Q0ZHG (*P* < 0.01, Figure 4B).

In the DRG 4 weeks post vector injection, there was no significant increase in SDF1α in neuropathic rats with Q0ZHG compared with that in the sham rats (Figure 4C), however, the expression of SDF1α in neuropathic rats treated with QHIL10 was lower than that in neuropathic rats with Q0ZHG (*P* < 0.05, Figure 4C). In the SDH samples 4 weeks post vector injection, neuropathic rats with Q0ZHG showed a statistically insignificant increase in the expression of SDF1α compared with sham rats (*P =* 0.07*,* Figure 4D); the expression of SDF1α in neuropathic rats treated with QHIL10, was markedly lower than that in neuropathic rats with Q0ZHG (*P <*0.05*,* Figure 4D).

### The effect of the HSV vectors over-expressing IL-10 on CXCR4 in the DRG and the SDH in the gp120 + ddC model

**Figure 5:**
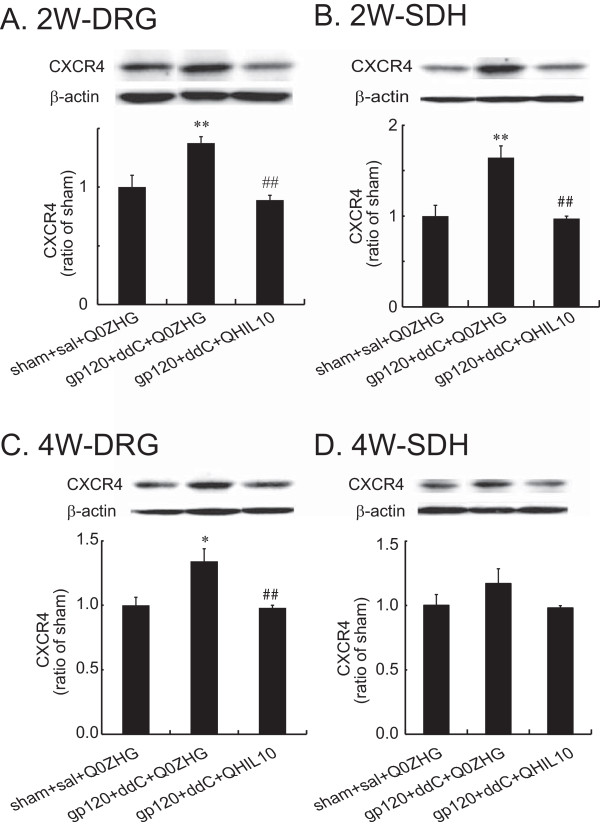
**The effect of IL-10 mediated by the HSV vectors on the expression of CXCR4 in the DRG and the spinal cord at 2 or 4 weeks.** Rats with neuropathic pain were inoculated with QHIL10 or Q0ZHG 1 week post gp120 with ddC. In the control group, rats received the sham surgery with saline IP injection and Q0ZHG (sham + sal + Q0ZHG). The data were analyzed using one way ANOVA with *post hoc* PLSD test, mean ± SEM. **(A and B)** Two weeks post vector injection, the L4/5 DRG **(A)** and the SDH **(B)** were harvested, and the expression of CXCR4 was tested using western blots. **P < 0.01 *vs.* control, ## *P* < 0.01 *vs* gp120 + ddC + Q0ZHG, n = 4 rats. **(C and D)** Four weeks post vector injection, the L4/5 DRG **(C)** and the SDH **(D)** were harvested, and the expression of CXCR4 was tested using western blots. *P < 0.05 *vs* control, ## *P* < 0.01 *vs.* gp120 + ddC + Q0ZHG, n = 4 rats.

In the DRG 2 weeks post vector injection, neuropathic rats inoculated with Q0ZHG showed a statistically significant increase in CXCR4 compared with the sham (*P* < 0.01, Figure 5A); the expression of CXCR4 in neuropathic rats with QHIL10, was significantly lower than that in neuropathic rats with Q0ZHG (*P* < 0.01, Figure 5B). In the SDH, there was a significant increase in CXCR4 in neuropathic rats with Q0ZHG compared with sham rats (*P <* 0.01, Figure 5B); CXCR4 in neuropathic rats with QHIL10, was markedly lower than that in neuropathic rats with Q0ZHG (*P <* 0.01, Figure 5B*).*

In the DRG 4 weeks post vector injection, CXCR4 was significantly higher in neuropathic pain rats with Q0ZHG than that in sham rats (*P* < 0.05 *vs* sham + sal + Q0ZHG, Figure 5C); the increased expression of CXCR4 in neuropathic rats with QHIL10, was lower than that with Q0ZHG (*P* < 0.01 *vs* gp120 + ddC + Q0ZHG, Figure 5C). There was no significant difference in neuropathic rats with Q0ZHG compared with either sham rats or neuropathic rats with QHIL10 (Figure 5D) in the SDH samples at 4 weeks after vector injection.

## Discussion

The chronic nature of neuropathic pain can dramatically reduce productivity and quality of life, and is notoriously difficult to manage using currently available therapeutic regimens. The principal immunomodulatory activity of IL-10 is to inhibit the production of proinflammatory cytokines [21]. We have previously demonstrated that IL-10 mediated by the HSV vectors reversed formalin-induced inflammatory pain [8]. Recent studies show that animals inoculated with the HSV vectors expressing IL10 reduces mechanical allodynia induced by the spinal cord injury [27]. Previous studies have also demonstrated that the application of gp120 onto sciatic nerve, systemic ddC, or the combination of these treatments, resulted in NP characterized by mechanical allodynia and upregulation of inflammatory factors in the spinal cord and/or the DRG [4,15,29-31]. Moreover, these effects can be inhibited by intrathecal anti-inflammatory chemicals or by HSV-mediated TNF soluble receptor [14,17]. The current study demonstrates that gp120 combined with ddC induced neuropathic pain, and that IL-10 mediated by the HSV vectors resulted in a significant elevation of mechanical threshold that was apparent one week post vector inoculation. Specifically, AUC of the mechanical threshold response in the HSV vectors expressing IL-10 was increased compared with the control vectors, indicating the markedly anti-allodynic effect of IL-10. The HSV vectors expressing IL-10 reversed the upregulation of p-p38, TNFα, SDF1α, and CXCR4 induced by gp120 with ddC in the lumbar SDH and the DRG at 2 and/or 4 weeks.

MAPKs such as p38 are important for intracellular signal transduction and play critical roles in regulating neural plasticity and inflammatory responses [34]. The signaling of p38 is critical upon exposure to HIV gp120 for the neurotoxic phenotype of monocytic cells [35,36]. In *in vivo* studies, Milligan and colleague have reported that the systemic p38 inhibitor CNI-1493 blocks intrathecal gp120-induced mechanical allodynia [37]. Our unpublished data show that systemic ddC induced the upregulation of p-p38 in the spinal cord dorsal horn and the DRG.

HIV virus infection is able to increase the production of several cytokines [38]. It is reported that there is an increased level of TNFα in the CSF [39], blood plasma [40], spinal cord [41], and brain [42] in patients with HIV. We and others have reported that the application of the recombinant gp120 to the sciatic nerve increases TNFα in the DRG and the spinal cord [4,28]. Furthermore, intrathecal TNFα siRNA or TNF soluble receptor (TNFSR) reduces the gp120 application-induced mechanical allodynia, indicating that TNFα in the spinal cord and/or the DRG are involved in neuropathic pain induced by HIV gp120 [4]. TNF soluble receptor mediated by the HSV vectors suppresses gp120-induced neuropathic pain and reduces TNFα [14]. Taken together, these data highlight the importance of TNFα in the development of the exaggerated pain state related to HIV gp120.

HAART has dramatically reduced the HIV/AIDS associated morbidity and mortality [43]. Although the incidence of most neurological complications of HIV has fallen with HAART, rate of HIV-SN has been rising [44]. One of the reasons is that NRTIs are neurotoxic and can cause a dose-dependent painful peripheral neuropathy [45]. Our previous studies demonstrate that TNFα is involved in the mechanisms of ddC-induced neuropathic pain [30]. Knockdown of TNFα with siRNA blocks the mechanical allodynia induced by ddC; intrathecal administration of recombinant TNFSR, reverses mechanical allodynia induced by ddC, suggesting that TNFα is involved in ddC-induced neuropathic pain [30]. Using HSV vectors expressing TNFSR, we extended our previous studies and found that it suppressed mechanical allodynia and decreased TNFα induced by ddC [17].

Evidence shows that chemokines and their receptors play an important role in inducing and maintaining neuropathic pain [14-16]. Chemokine receptors, in particular CXCR4 and CCR5, mediate HIV infection of immunocompetent cells as well as microglia [11]. The interplay of TNFα and HIV-1 leads to the enhanced expression of toxic chemokines [46]. CXCR4 and its ligand SDF1α are important factors in the neuropathogenesis of HIV/AIDS [11]. HIV gp120 may bind to and activate CXCR4 expressed in the DRG neurons in a CD-4-independent manner [47,48], suggesting the direct neurotoxic effects of gp120 on the neurons [49]. Our recent studies have shown that HIV gp120 induces the upregulation of SDF1 and CXCR4 in the spinal cord and the DRG [14]. White and colleagues reported that systemic ddC induces the expression of CXCR4 mRNA in glia and neurons, and SDF1 mRNA in glia [15]. Pain hypersensitivity produced by ddC is inhibited by systemic CXCR4 antagonist, AMD3100, suggesting that NRTIs produce painful hypersensitivity through the CXCR4 signaling in the DRG [15]. We report that ddC induces the overexpression of SDF1α and CXCR4 in the protein level in the spinal cord and the DRG, and that intrathecal administration of AMD3100 reverses the mechanical allodynia induced by ddC [17]. In *in vitro* studies, SDF1 is produced under the control of inflammatory factors, such as TNFα [50]. TNFα significantly enhances expression of CXCR4, which facilitates the chemotactic invasiveness of human mesenchymal stem cells toward SDF1α [33]. Our studies demonstrate that blockage of TNFα by HSV-mediated TNFSR reverses the upregulation of SDF1α and CXCR4, suggesting that SDF1α/CXCR4 system is involved in the mechanisms of TNFα in neuropathic pain induced by gp120 or ddC [14,17].

IL-10 inhibits the production of proinflammatory cytokines [21,22]. In the *in vitro* studies, IL-10 diminishes TNF mRNA after the onset of stimulation of polymorphonuclear leukocytes with LPS, identifying the biological action of IL-10 as a suppressor of the inflammatory response [21]. We have shown that IL-10 reduces the p-p38 and decreases the expression of full-length membrane spanning TNFα following lipopolysaccharide stimulation of microglia *in vitro*; IL-10 also reduces intracellular cleavage of membrane TNFα [8]. Hypoxia-mediated increases in CXCR4 expression and cell survival are lower in IL-10-deficient othelial progenitor cell [51]. IL-10 also downregulates CXCR4 mRNA expression in CD4^+^ T lymphocytes [52]. In the *in vivo* studies, IL-10 inhibits the writhing response induced by acetic acid or zymosan in mice, and the knee joint incapacitation induced by zymosan in rats; IL-10 inhibits the release of TNFα from mice peritoneal macrophages obtained after local injection of zymosan [53]. Acute intrathecal administration of rat IL-10 protein itself briefly reverses CCI-induced mechanical allodynia [54]. Hyperalgesic responses to TNFα or carrageenan are inhibited by intraplantar administration of IL-10 [55]. In the present studies, we report for the first time that IL-10 suppresses TNFα and SDF1/CXCR4 in the neuropathic pain state induced by gp120 with ddC.

To produce a long-term analgesic effect, non-viral plasmids or viral vectors expressing IL-10 may represent a promising approach in a variety of pain states. Intrathecal delivery of plasmid DNA encoding IL-10 gene prevents, and progressively reverses the allodynic state induced by paclitaxel (a chemotherapy drug), and markedly decreases paclitaxel-induced expression of TNF mRNA in the lumbar DRG [56]. Repeated intrathecal delivery of the plasmid DNA vectors encoding IL-10 gene abolishes neuropathic pain induced by sciatic chronic constriction injury (CCI) [23]. Adenoviral vectors encoding human IL-10 gene prevent and reverse thermal hyperalgesia and mechanical allodynia in the CCI model [54]. Gene transfer to the primary sensory neurons of the DRG with self-complementary recombinant adeno-associated virus serotype 8 expressing IL-10, leads to significant reversal of mechanical allodynia in chronic neuropathic pain induced by L5 spinal nerve ligation [57]. We have found that transduction of the DRG neurons *in vivo* achieved by subcutaneous inoculation of the HSV vectors in the foot results in production of transgene-coded IL-10 in the DRG neurons and transport of the gene product to terminals in the spinal cord, suppresses the formalin-induced nociceptive effect and reduces TNFα and p-p38 expression [8]. IL-10 mediated by HSV almost totally reversed the upregulation of mRNA of TNFα in the spinal cord in the formalin pain model [8]. Recent studies show that animals inoculated with the HSV vectors expressing IL10 reduces mechanical allodynia induced by the spinal cord injury, which correlates with a significant decrease in spinal TNFα [27]. In the current studies, we extend our previous results showing that IL-10 expressed by the HSV vectors reduced neuropathic pain induced by HIV gp120 combined with ddC, and reversed the upregulation of p-p38, TNFα, SDF1α, and CXCR4 in the neuropathic state in the lumbar SDH and the DRG at 2 and/or 4 weeks. The mechanisms by which IL-10 reduces neuropathic pain are not clear. Previous studies suggest that TNFα mediated SDF1α/ CXCR4 pathway in the gp120 and ddC induced neuropathic pain models [14,17]. It is possible that in this study, IL-10 suppressed SDF1α/ CXCR4 through reduced TNFα signaling in the gp120 combined with ddC state. Local application of gp120 to sciatic nerve induces wide neurochemical changes in both the DRG and the spinal cord. Meanwhile, IL-10 mediated by HSV reduced those inflammatory factors in both the DRG and the spinal cord. It is possible that IL10 may protect against HIV-induced pain by preserving integrity of gene expression in DRG and thus preventing abnormal release of nociceptive peptides from DRG neurons into the dorsal horn. Future work will study the exact molecular mechanisms/pathways by which IL-10 suppresses those inflammatory factors.

## Methods

### A non-replicating HSV-based vectors expressing IL10

The construction of HSV vectors expressing IL10 has been described (designated in that report as QHIL10) [58]. QHIL10 contains the full-length rat IL10 gene tagged with hemagglutinin (HA) under the control of the human cytomegalovirus immediate-early promoter (HCMC IEp); the control vectors contains the *lacZ* gene (Q0ZHG) in place of *IL10*-HA [27]. In our previous studies, we demonstrated that the vectors produce IL10 from the primary DRG neurons infected *in vitro* and in the spinal cord *in vivo*[8]. The investigators of vector injection were blinded for behavior testing.

### Animals

Male Sprague-Dawley rats weighing 225 to 250 g were housed 1 to 3 per cage approximately 7 days prior to the beginning of the study. Rats were maintained with free access to food and water and were on a 12:12, light:dark schedule at 21°C and 60% humidity. All housing conditions and experimental procedures were approved by the University Animal Care and Use Committee at the University of Miami, FL., and were conducted in accordance with the ethical guidelines of the International Association for the Study of Pain.

### Model of neuropathic pain induced by HIV gp120 combined with systemic ddC

Under 1-2% isoflurane anesthesia and aseptic surgical conditions, the left sciatic nerve of rats was exposed in the popliteal fossa without damaging the perineurium. The sciatic nerve was wrapped loosely, with a strip of oxidized regenerated cellulose (Surgicell, Ethicon), previously soaked in 200 μl of a 0.1% rat serum albumin (RSA) in saline solution, containing 40 ng of gp120-MN (Immunodiagnostics, Bedford, MA). The procedure was performed on sham-operated animals but without application of gp120 as previously reported [14,28]. The nerve was gently manipulated back into place and incisions of skin closed with staples. Rats were injected with 1 ml of ddC (20 mg/kg, IP) [31] at the time of application of perineural gp120 (henceforth referred to as gp120 + ddC) as previously reported [29]. Sham controls were treated with perineural RSA and IP saline in the same regime as perineural gp120 and systemic ddC treatment.

### Mechanical threshold

Animals were placed in non-transparent plastic cubicles on a mesh floor for an acclimatization period of at least 30 min on the morning of the test day. Mechanical threshold was determined by assessing paw withdrawal to von Frey filaments (Stoelting, Wood Dale, IL) of graded tensile strength. A series of calibrated von Frey filaments were presented serially to the hind paw in ascending order of strength, with each filament applied for 6 s with sufficient force to cause slight bending against the paw. A positive response was defined as a rapid withdrawal and/or licking of the paw immediately on application of the stimulus. Whenever a positive response to a stimulus occurred, the next smaller von Frey hair was applied, and whenever a negative response occurred, the next higher force was applied. In the absence of a response at a pressure of 15.1 g, animals were assigned to this cutoff value. The tactile stimulus producing a 50% likelihood of withdrawal was determined using the up-and-down method [3].

### Western blots

Under deep anesthesia, the L4-5 DRG or the spinal cord was removed rapidly, frozen on dry ice, and stored at -80°C. These tissues of the spinal dorsal horn dissected following the spinal cord samples were homogenized in protein lysis buffer (150 mM sodium chloride, 1.0% NP-40, 0.5% sodium deoxycholate, 0.1% SDS, 50 mM Tris, pH 8.0) containing protease inhibitors and phosphatase inhibitors (Phosphatase Inhibitor Cocktails 1/2). The homogenate was centrifuged at 18,000 g for 20 min at 4°C. The supernatant was collected and assayed for protein concentration using the DC protein assay (Bio-Rad). Aliquots containing 30 μg of protein were dissolved in Laemmli buffer and denatured at 95°C for 5 min; the proteins were separated by 10% Tris-glycine SDS-PAGE gel and transferred to a PVDF membrane. The membranes were blocked with 5% nonfat dry milk in PBS buffer, and then incubated with primary antibodies overnight at 4°C, including rabbit anti-p-p38 (1:1000, Santa Cruz Biotechnology, Santa Cruz, CA), rabbit polyclonal anti-TNFα (1 : 500, Chemicon, Temecula, CA), goat anti-CXCR4 (1:1000, Santa Cruz Biotechnology), rabbit anti-SDF1a (1:500, ABCAM, Cambridge, MA), and mouse anti-β-actin (1 : 8000, Sigma). The blots were incubated with secondary antibodies (Santa Cruz Biotechnology), and developed in chemiluminescence solution (Pierce Biotechnology). Quantification of Western blots was done from the obtained chemiluminescence values (BioRad ChemiDoc). Target protein bands were normalized using the amount of β-actin.

### Data analysis

The statistical significance of the differences of neurochemical changes was determined by the *t* test or one-way ANOVA *post-hoc* test following Fisher’s PLSD (StatView5). To compare the difference between the time-course curves of the behavioral testing we used repeated measures ANOVA with one within-subjects factor (time) and one between-subjects factor (group) of a General Linear Model (IBM, SPSS21). All data were presented as mean ± SEM, and *P*-values of less than 0.05 were considered to be statistically significant.

## Competing interests

The authors declare that they have no competing interests in the work.

## Authors’ contributions

WZ, WH, SH conceived and designed the study. WZ, WH, SL, and SH performed experiments and analyzed data. WZ, WH, RCL, KAC, DAL and SH wrote the manuscript. All authors have read and approved the final manuscript.
